# The Institutional Library

**Published:** 1914-05-02

**Authors:** 


### THE INSTITUTIONAL LIBRARY.

### The Path to Promotion.

The Foundations of Character. Being a Study of the Tendencies of the Emotions and Sentiments. By Alexander F. Shand, M.A. (Macmillan and Co. Price 12s. net.)

This delightful book ought to be read by every institutional officer who has to control and manage other people, for it provides the *rationale* of the habits of mind, of the tact, and of the resourcefulness which are the, generally quite subconscious, means which in their successful employment lead a man to be called a capable administrator. It is true that, for the most part, such men pride themselves on being "practical" and use the complementary adjective "theoretical" with a certain condescension of contempt. Such men delight to flatter themselves that their success is due to "character" or "personality," and are content to stop there. Nevertheless if a man is pledged by his calling, as is the institutional administrator for instance, to fight against lazy habits of body and slovenly methods wherever he finds them in those under his control, there is no excuse for him to be content with lazy habits of mind in himself. And to ascribe his success or influence to his "character" is mere intellectual laziness. Mr. Shand has, therefore, contributed to psychology an interesting analysis of the constituents of character, which he finds, as we expect him to find, to be a complex result to which a variety of inherited and acquired factors contribute; which obeys certain laws, and which, in short, is capable of being understood in its working. For character by derivation, as the Greeks knew, is simply the hallmark, the convenient stamp, by which we agree to sum up an individual's qualities, knowing that were such short-cuts and convenient generalisations abandoned in the supposed interests of exact speech we should never be able to complete a single sentence of conversation. Personality, again, is the mask or trade-mark which we use conveniently to sum up a bundle of traits in the individual. While, therefore, no institutional officer will learn how to administer an institution after reading this work—just as no amount of successful organising could have enabled him to write it—yet the man who has succeeded in running his department, or to whom some degree of responsibility is given, can gain much value from its study because the reading of it will make plain to him the laws and methods of the process which he applies every day as it were by instinct. As Mr. Shand well says in his chapter devoted to "instinct and emotion," the animal with the more rigid instincts has the advantage at birth over one so much dependent on experience if both are left to their own resources; the other has an incalculable advantage afterwards." For "animal" read "hospital officer," for "at birth" read "on appointment," and for "afterwards" substitute "after reading this work," and the gain to the practical man by its study becomes apparent. For the practical man forgets that to the man of affairs thought, study, and theory are as much *experience* as actually carrying ideas into practice are experience to the armchair critic. While it is impossible here to go through this volume book by book and chapter by chapter, and the fluent ease of the writer's style really holds out that invitation to the reviewer, a few words of criticism on Mr. Shand's treatment of "instinct and emotion" may be added. Where many definitions of instinct are quoted and many writers referred to, pointing thus to a lively scholarship in the writer, no mention, we are surprised and regret to see, is made of the witty and sainted Samuel Butler's contribution to the subject, which, in fact, he made his own. For example, Mr. Shand defines instinct (page 182) as "an inherited disposition both to be excited by certain stimuli and to respond with a specific kind of behaviour or expression to such stimuli." He also defines (page 180) instinctive as "the capacity which is inherited to perform certain complex actions or behaviours characteristic of a group of animals." Samuel Butler defined instinct as "inherited memory," and if the best definition is that which gives us the essential qualities of the object defined with nothing unessential added, Butler's definition is clearly the best. Its terseness, of course, is the proof of it. Surely a man of such evident breadth of reading cannot have missed "Life and Habit" and "Unconscious Memory," the first and third volumes of that inspiring tetralogy which has placed modern science under so great a debt?

To sum up, the institutional manager who reads this volume not merely will be able to inform his committee or authorities that So-and-so is capable or the reverse, but he will be provided with a key to unlock the secrets of why he or she is so. It ought to be invaluable to the writer of testimonials and lend a new interest to these pious, colourless evasions of the truth. Finally, let no subordinate officer imagine that the work is not for him also. For if the superintendent has to manage the institution his subordinates have also to learn how to manage him. This work will help them to understand the process and bring down character from the clouds of thoughtlessness into the light of common sense and understanding. The path to promotion lies through the pages of this work.

### Speech and Sight for the Dumb and Blind.

Man's Miracle: The Story of Helen Keller and her European Sisters. From the French of Gerard Harry. (William Heinemann. Price 3s. 6d. net.)

The metamorphosis of Helen Keller, the blind, deaf, and dumb American girl, who became not merely "sensible to sight as to feeling," to misquote Shakespeare, but a woman of such education, learning, and culture as to earn from Mark Twain the eulogy that "the nineteenth century has produced two exceptional individuals—Napoleon and Helen Keller," is the subject of this book. Nor can an undistinguished style detract from its interest, which is, in fact, threefold. First, in appeal to the ordinary reader, comes the fact that a human being to whom apparently all the avenues of sense had been closed from birth became as if they had always been open, became, in fact, a finer woman owing to the stimulus of the enormous difficulties which she surmounted; secondly, to the hospital world, is the triumph of the scientific method of which her life becomes the living record; and, lastly, and most interesting in a human sense of all, the story of the supreme patience, endurance, and courage of Miss Anna Sullivan, by whose means the clogged senses were set free. Truly the idealists, whether in art, medicine, or religion, are the altruists and egotists *par excellence*. They combine the virtues of both: the altruist in his self-sacrifice and the egotist in his disregard of others. If the scientist sacrifices his energies to science, he generally sacrifices everyone who comes in personal contact with him to science also. The wives of men of genius in all departments remain to show that this is true, and whether Miss Sullivan in her heroic struggle to make the dumb speak and the deaf to hear actually did so or not is beside the question, since she belongs to the type in which egoism and altruism are combined. If interest in subject-matter is enough to make a book read, this book should have as wide a circulation as, say, Robert Blatchford's "Merrie England." If it does not meet with the same success, then we shall be forced to conclude that a book must not only be interesting but readable—and there is no room in this review for a discussion of style.

### A Handbook of Hygiene.

First Stage Hygiene. By Robert A. Lyster, M.D., D.P.H. (London: W. B. Clive, University Tutorial Press, Ltd. Price 2s. 6d.)

The sixth edition of this volume bears witness to the fact that the author has succeeded in his aim not merely to present a medley of facts on the subject, but "to treat the successive points in logical order, and to give unity to the subject." The order he has chosen begins with a chapter on "The General Build of the Body," treats separately the blood, respiration, ventilation, foods, the digestive system; cooking; beverages; the spleen; the skin, with soap and cleanliness; the nervous system; personal hygiene, clothing; accidents and emergencies; soils, sites, and climates; the water supply; heating the dwelling-house; and the removal of house refuse. This is an instructive list, and it concludes with some test questions and specimen examination papers. If an author on such a subject and in such a series can avoid the twin difficulties of being too elementary to be of value, and too technically learned, his readers have much to be grateful for. And in this case their gratitude will not be misplaced.

Applied Bacteriology for Nurses. By Charles F. Bolduan, M.D., and Marie Grund, M.D. Illustrated. (Philadelphia and London: W. B. Saunders Company. 1913. Pp. 160. Price 5s.)

That a nurse should know at least some of the fundamental principles and the commonest details of the now all-pervading science of bacteriology is a reasonable proposition, and if she can assimilate such knowledge without scamping the practice of her real function, there is nothing to be said against excursions into a sphere beyond her province. But we object entirely to the view that such "understanding is an indispensable part of every nurse's mental equipment." However, as far as this manual is concerned, it must be granted that the authors have presented the main facts of elementary bacteriology in a manner suitable to the class of reader catered for. It is gratifying to note that even in America it is not considered necessary that nurses should do individual laboratory work, and demonstrations by the teacher of practical work with cultures are deemed sufficient.

### A Pompous "Symposium."

Youth. Edited by T. N. Kelynack, M.D. National Health Manuals. (London: C. H. Kelly. 1913. Pp. 152. Price 1s. net.)

This symposium is a continuation of a series designed to instruct those "interested or engaged in social service"; the volumes upon infancy, childhood, and school life have already been published. Each chapter deals with a particular aspect of the subject, and is by a different author, usually a well-known medical expert. They are, however, so uniformly pompous and platitudinous that upon purely internal evidence one would never have suspected a diverse authorship. To each chapter is prefixed a page of quotations from Biblical and more modern poetry and prose. The sentiments expressed in these passages are admirable, but we are not sure that it was really worth the trouble of printing them. The various contributors balance themselves upon fences with much dexterity.

Diseases of the Ear, Nose, and Throat. By George Nixon Biggs, M.B., B.S. (Durh.). Illustrated. (London: University of London Press and Hodder and Stoughton. 1914. Price 10s. 6d.)

The series of text-books issued under the name of the London Medical Publications already contains several volumes which should appeal strongly to general practitioners, and the latest addition to this series is likely to rank among the first five in popularity. Should any general practitioner desire to take up nose, throat, and ear work, he will find no difficulty in obtaining a good grounding in the details of this practice from Mr. Biggs' new manual. To understand what is to be done and why it should be done in such and such a manner is naturally a good start towards successful accomplishment with one's own hands. Intending operators will hardly find a book with more lucid diagrams than those in this volume, while their frequency enables the steps of any procedure to be followed with a facility as nearly equal as possible to that of actual individual tuition. But, besides operative details, the book describes fully and dogmatically diagnosis, treatment, symptoms, etc., all *secundum artem*; practitioners who have no intention of operating can easily assimilate the cardinal truths of this branch of practice, and thus be in a position to offer timely and authoritative warnings to their patients.

### A Credit to British Surgery.

The Surgery of the Stomach. By H. J. Paterson, M.C., F.R.C.S. Second Edition. (J. Nisbet and Co. 1914. Pp. 342. Price 15s. net.)

This text-book is well produced and well compiled, and is a credit to British surgery; it is not surprising that a second edition should be called for so soon after the first one. Mr. Paterson's dogmatic style makes his teaching emphatic and impressive; but there are, of course, other views held upon some of the matters which he discusses. His faith in the analysis of test meals as aids to diagnosis, for instance, is much greater than that of a good many surgeons, though we should be sorry to say that it is misplaced on that account. The monograph is so complete that omissions are hard indeed to find. One small point upon which Mr. Paterson's opinion would be interesting, if he would give it in the next edition, is this: Does he subscribe to the view that reversed peristalsis in the stomach, as demonstrated by bismuth meal and radiography, is indicative of non-malignant conditions only, and is never seen when there is cancer of the stomach?

How to Diagnose Smallpox. By W. McC. Wanklyn. Illustrated. (London: Smith, Elder and Co. 1913. Demy 8vo. Price 3s. 6d. net.)

This book is a good representative of that class known as "easy readers." It is written mostly in a distinctly "chatty" style, and thus the theme has been made to spread to well over one hundred pages. Dr. Wanklyn is one of a comparatively small band who have had in this country a considerable experience of smallpox, and he is doing a useful service in adding a convenient manual on the subject to the existing literature. As to his choice of style we feel some doubts. While his treatise on diagnosis is delightful reading and leaves nothing to be wished for on the score of dogmatic sufficiency, yet it is too long-winded for use by a general practitioner in a time of emergency. Nevertheless the book is a useful exposition of the difficulties and traps which seem such a serious factor in the recognition of this disease.

